# The association of bullous pemphigoid with dipeptidyl-peptidase 4 inhibitors: a ten-year prospective observational study

**DOI:** 10.1186/s12902-021-00689-7

**Published:** 2021-02-11

**Authors:** Vaia Lambadiari, Aikaterini Kountouri, Foteini Kousathana, Emmanouil Korakas, Georgios Kokkalis, Sofia Theotokoglou, Lina Palaiodimou, Pelagia Katsimbri, Ignatios Ikonomidis, Konstantinos Theodoropoulos, Evangelia Papadavid

**Affiliations:** 1 Second Department of Internal Medicine Research Unit and Diabetes Center, Attikon University Hospital, Medical School, National and Kapodistrian University of Athens, Rimini 1 Chaidari, Athens, Greece; 2 Second Department of Dermatology and Venereology, Attikon University Hospital, Medical School, National and Kapodistrian University of Athens, Athens, Greece; 3 Second Department of Neurology, Attikon University Hospital, Medical School, National and Kapodistrian University of Athens, Athens, Greece; 4 Fourth Department of Internal Medicine, Attikon University Hospital, Medical School, National and Kapodistrian University of Athens, Athens, Greece; 5 Second Cardiology Department, Attikon University Hospital, Medical school, National and Kapodistrian University of Athens, Athens, Greece

**Keywords:** Bullous pemphigoid, Dipeptidyl-peptidase 4 inhibitors, Vildagliptin, Gliptins

## Abstract

**Background:**

Bullous pemphigoid is the most common bullous chronic autoimmune skin disease. Recent studies have suggested dipeptidyl-peptidase 4 inhibitors as possible predisposing agents of bullous pemphigoid. The objective of our study was to prospectively estimate the association between gliptins and the development of bullous pemphigoid.

**Methods:**

We conducted a prospective study which included all patients diagnosed with biopsy-proven bullous pemphigoid in the Dermatology Department of our hospital between April 1, 2009 and December 31,2019. The diagnosis of bullous pemphigoid was based on specific clinical, histological and immunological features.

**Results:**

Overall 113 consecutive patients (age 75 ± 13 years, 62 females) with the diagnosis of bullous pemphigoid were enrolled. Seventy-six patients (67.3%) suffered from type 2 Diabetes and 52 (46%) were treated with dipeptidyl-peptidase 4 inhibitors. The most frequent prescribed gliptin was vildagliptin, being administered to 45 cases (39.8% of total patients enrolled, 86.5% of the patients treated with gliptins). Gliptins were withdrawn immediately after the diagnosis of bullous pemphigoid, which together with steroid administration led to remission of the rash.

**Conclusions:**

This study revealed that treatment with dipeptidyl-peptidase 4 inhibitors, especially vildagliptin, is significantly associated with an increased risk of bullous pemphigoid development.

## Background

Bullous pemphigoid (BP) is the most common chronic bullous autoimmune skin disease which is characterized by the presentation of subepidermical blisters. It is considered as a disease of the elderly people and is associated with significant morbility and mortality [1]. The pathogenesis of BP is characterized by autoimmune response against hemidesmosomal protein (BP180, BP320) at the dermoepidermal junction. Although the etiology of BP is unknown an increased number of neurologic and psychiatric disorder have been described to raise the risk of BP. Furthermore, various drugs (spironolactone, furosemide, antibiotics) have been reported as possible risk factors for the development of BP. Recent studies have suggested that dipeptidyl peptidase-4 inhibitors (DPP4-is), an incretin-based drug for type 2 diabetes, as possible predisposing agents of BP [2, 3]. DPP4-is inhibit the degradation of incretins resulted in the improvement of blood glucose levels by increasing insulin secretion and decreasing of glucagon secretion. The objective of our study was to estimate the association between the use of DPP4-is and the development of BP in the setting of a tertiary university hospital and to raise awareness for everyday clinical practice, both among dermatologists as well as all physicians following patients with diabetes.

## Methods

### Design and study population

The study was designed as an observational prospective study. In our study included all patients who received a new diagnosis of BP and hospitalized in the Dermatology Department between April 1, 2009 and December 31, 2019.Inclusion criteria for the study were the diagnosis of new-onset BP (presentation of BP the last 4 months) and severe/extensive BP requiring hospitalization in the dermatology department. Exclusion criteria were: οther forms of pemphigoid (non-bullous), malignancy and patients under treatment with DPP4-is for more than 2 years were exclusion criteria. Diagnosis of BP was based on the following criteria: a) compatible clinical characteristics including urticarial, eczematous, excoriated and/or bullous lesions associated with variable itch b) subepidermal clefts, eosinophilic spongiosis, and/or dermal infiltration of eosinophils in specimen from the edge of the blister as assessed by histopathologic analysis c) deposition of IgG and C3 in the linear band on at dermal-epidermal junction of perilesional skin by direct IF microscopy. All patients have been inadequately treated with topical corticosteroids as a primary regimen and therefore admitted to the tertiary hospital for optimal management with systemic corticosteroids, with the addition of immunosuppressant medications, namely azathioprine, in some cases. We recorded medical data including patients’ age, sex, comorbidities, concomitant medications. This study was approved by the institutional ethical board of Attikon University hospital. A signed informed consent was obtained from all subjects prior to any procedure included in the study protocol. All methods were carried out in accordance with relevant guidelines and regulations. (Declaration of Helsinki)

### Statistical analysis

The mean and standard deviation for continuous variables and the number and percentage of patients within each categorical variable were used. The association between the use of DPP4-is and the severity of BP was evaluated in univariate and multivariate logistic regression analysis. For multivariate analysis variables with *p* < 0.1 at univariate analysis or with clinical significance were used. A value of Bullous Pemphigoid Disease Area Index (BPDAI) greater than 56 was used as a measure of severe BP as previously published [4] (This cut off value was identical with the median value of BPDAI in our study cohort).

The Odds Ratio (OR) and the respective 95% confidence intervals (CIs) were calculated. A *p* value< 0.05 was considered statistically significant. Data analysis was performed using IBM SPSS V23.

### Clinical outcomes

We reviewed the percentage of patients with type 2 diabetes among all patients with BP. The number of patients who were under treatment with DPP4-is and the specific type of DPP4-is prescribed (vildagliptin, sitagliptin, linagliptin, saxagliptin and alogliptin) were also examined. We also assessed other comorbidities and co treatments. The effect of different types of treatment (systemic corticosteroids, immunosuppressive agents) on BP outcome was also evaluated.

## Results

### Demographic characteristics

Overall, 113 consecutive patients with the diagnosis of BP were enrolled in the study. The mean (SD) age was 75(13) years, and 62 patients were female (54.9%).

### The prevalence of DPP4-is and DM among the patients

Seventy-six patients (67.3%) suffered from type 2 Diabetes Mellitus. The mean (SD) age was 75(11) years, and 41 patients were female (54%). There were no differences in age and sex between diabetic and non-diabetic patients.

Fifty-two (46%) were under treatment with DPP-4is at the onset of BP. The mean (SD) age was 74 (10.17) years, and 23 patients were female (44.23%). The mean (SD) time between the initiation of DPP4-is administration and the onset of the skin lesions was 10 ± 2 months. The most frequent prescribed DPP4-i was vildagliptin, being administered to 45 cases (39.8% of total patients enrolled, 86.5% of the patients treated with DPP4-is). Three patients (2.7%) were treated with linagliptin, three (2.7%) with sitagliptin, one (0.9%) with alogliptin and one (0.9%) with saxagliptin (Table 1).

**Table 1:** Percentage of patients with Bullous pemphigoid and diabetes mellitus according to the type of gliptins

	n	% patients with BP	% patients with type 2 diabetes
Patients with diabetes	76	67.3%	100%
Treatment with DPP-4is	52	46%	68%
Vildagliptin	11	9.7%	14.4%
Vildagliptin-Metformin	34	30.1%	44.73%
Linagliptin	3	2.7%	3.9%
Sitagliptin	1	0.9%	1.3%
Sitagliptin-Metformin	2	1.8%	2.6%
Alogliptin-Metformin	1	0.9%	1.3%
Saxagliptin	1	0.9%	13%

*BP* Bullous pemphigoid, *DPP-4is* Dipeptidyl-peptidase 4 inhibitors

### Concomitant medications possibly associated with BP

No possible association of the patients’ concomitant medications and BP was found, except for two (1,7%) patients receiving spironolactone, three (2,6%) patients receiving levodopa and one patient receiving biperiden. However, as the patients had been for at least 3 years on these regimens, any possible causal relationship between the drugs and the BP was rejected.

### The association of DPP4-is with bullous pemphigoid severity

By multivariate analysis after adjustment for age and sex, the odds ratio of DPP4 use for severe BP (BPDAI> 56) was OR: 2.97, 95% CI = 1.24–7.02, *p* = 0.014. Female sex was associated with BP severity with OR: 2.88, 95% CI = 1.20–6.89, *p* = 0.017 in multivariate analysis. By univariate analysis spironolactone, levodopa and biperiden were not associated with the development of severe BP (BPDAI> 56), (*p* > 0.1 for all associations).

### Rash remission-treatment

DPP4-is were withdrawn immediately after the diagnosis of BP. 92 (81.4%) patients were treated with systemic corticosteroids and 14 patients (12.4%) received azathioprine as an add-on therapy to systemic corticosteroids. Prednisolone was administered in 80 (70,8%) cases and methylprednisolone was administered in 12 (10,6%) cases. Complete remission of the rash was observed in all cases. The mean (SD) time for the remission of the rash was 15 ± 5 days. The remission was sustained after the definitive withdrawal of DPP-4is and no cases of relapse were reported.

## Discussion

This study suggests that treatment with DPP4-is, especially vildagliptin in this specific Greek population, are significantly associated with an increased risk of BP development. In addition, in our study we have shown that medication with DPP4-is was associated with BP severity as assessed by BPDAI score. The mechanism which is responsible for the development of BP has not yet been elucidated. It is known that DPP4 is a cell-surface glycoprotein with enzymatic activity that is expressed throughout the body including the skin. Various cell types, including keratinocytes and T cells, express DPP4, and its inhibition can increase the activity of numerous proinflammatory cytokines, leading to cutaneous eosinophil activation and blister formation [5]. Furthermore, DPP4 leads to the formation of plasmin, a major serine protease which cleaves the major BP autoantigen BP180; therefore, inhibition of DPP4 may lead to inappropriate cleavage and immunotolerance [6]. Association of BP with vildagliptin was significantly higher compared to the other DPP4-is. The reason for this is unclear, however, it has been suggested that the lower selectivity of vildagliptin for DPP4 compared to other DPP4-is leads to inhibition of DPP8 and DPP9 and a subsequent activation of the caspase-1 pathway, which plays a role in the pathogenesis of BP [7]. Furthermore, during the time that our study was conducted, vildagliptin had the highest market share among DPP4-is in Greece.

Since the introduction of DPP4-is as a standard treatment regimen for type 2 diabetes, numerous cases of patients developing BP following this treatment have been published [8, 9]. In accordance with the results of our study, vildagliptin has shown the strongest correlation in most reports [2, 10]. Skandalis et al. were the first who reported the risk of BP development in patients exposed to gliptins [11]. More recently, Kridin et al. found that DPP4-i exposure was associated with a 3-fold increased risk for BP, with the odds ratio (OR) being even higher for vildagliptin (10.7), and similar were the results in a study by Lee et al.*,* where the OR for developing BP under DPP4-i treatment was 1.58, with the highest adjusted OR being associated with the use of vildagliptin (1.81) [2, 10]. A significant limitation of our study is that it was not possible to establish whether DPP4-is per se or their combination with metformin may contribute to the development of BP. In all patients who received the combination DPP4-i/metformin, only the DPP4-i was discontinued. It is useful to mention, however, that there is no literature data implying an association between BP and metformin treatment. Furthermore, some of our patients were on medications that have been associated with BP. In the results we examined the percentage of patients who received drugs associated with BP based on metanalysis of Sian-De Liu et al. [12]. However, as the patients had been for at least 3 years on these regimens, any possible causal relationship between the drugs and the BP was rejected.

On the other hand, the main strength of our study is that has been designed as prospective study. To our knowledge is the first prospective study revealed the association between DPP4-is and BP. Another strength of our study is that it comprises a large number of patients with clinical and immunological diagnosis of BP who were exposed to DPP4-is, which is enough to establish an association between BP and these agents, especially vildagliptin. Moreover, as dermatologists and physicians treating people with diabetes rarely work together, and such cases may skip attention, we emphasize the importance of a multidisciplinary approach of such a population for early diagnosis, and the need to raise awareness for the contribution of DPP4-is to the development of BP with increasing prevalence.

## Conclusions

In conclusion, our study supports that there is a high risk of BP in patients exposed to DPP4-is. Further studies are warranted to determine the causal relationship between DPP4-is and BP development. Discontinuation of treatment with DPP-4 inhibitors was followed by remission of the rash, suggesting that such discontinuation should be immediately considered when BP is suspected. The increased exposure to these agents may be responsible for the increasing incidence of BP, especially in the elderly. The administration of DPP4-is needs to be carefully evaluated in high-risk patients.

## Data Availability

The datasets used and/or analysed during the current study are available from the corresponding author on reasonable request.

## Abbreviations

BP: Bullous pemphigoid
BP180: Bullous pemphigoid antigen 180
BPDAI: Bullous Pemphigoid Disease Area Index
CI: Confidence interval
DPP4: Dipeptidyl peptidase 4
DPP8: Dipeptidyl peptidase 8
DPP9: Dipeptidyl peptidase 9
DPP4-i: Dipeptidyl peptidase 4 inhibitor
OR: Odds Ratio
